# Non-Invasive Cerebellar Stimulation in Neurodegenerative Ataxia: A Literature Review

**DOI:** 10.3390/ijms21061948

**Published:** 2020-03-12

**Authors:** Alberto Benussi, Alvaro Pascual-Leone, Barbara Borroni

**Affiliations:** 1 Neurology Unit, Department of Clinical and Experimental Sciences, University of Brescia, 25123 Brescia, Italy; benussialberto@gmail.com; 2 Arthur and Hinda Marcus Institute for Aging Brain, Hebrew SeniorLife and Department of Neurology, Harvard Medical School, Boston, MA 02131, USA; apleone@hsl.harvard.edu; 3 Guttmann Brain Health Institute, Institute Guttmann, Universitat Autonoma, 08027 Barcelona, Spain

**Keywords:** transcranial magnetic stimulation, transcranial direct current stimulation, non-invasive brain stimulation, neurodegenerative ataxia, cerebellar ataxia, therapy

## Abstract

Cerebellar ataxias are a heterogenous group of degenerative disorders for which we currently lack effective and disease-modifying interventions. The field of non-invasive brain stimulation has made much progress in the development of specific stimulation protocols to modulate cerebellar excitability and try to restore the physiological activity of the cerebellum in patients with ataxia. In light of limited evidence-based pharmacologic and non-pharmacologic treatment options for patients with ataxia, several different non-invasive brain stimulation protocols have emerged, particularly employing repetitive transcranial magnetic stimulation (rTMS) or transcranial direct current stimulation (tDCS) techniques. In this review, we summarize the most relevant rTMS and tDCS therapeutic trials and discuss their implications in the care of patients with degenerative ataxias.

## 1. Introduction

Cerebellar ataxias encompass a heterogenous group of acquired and hereditary disorders with diverse clinical presentations. They manifest clinically with a combination of signs and symptoms, such as balance and coordination disturbances, oculomotor deficits, dysarthria, dysmetria, and kinetic tremor [1]. Both the hereditary and sporadic forms of neurodegenerative ataxia usually present in young adulthood [2], and have an estimated prevalence of 3 in every 100,000 people [3].

Most of the ataxias with a genetic origin are called spinocerebellar ataxias (SCAs), with more than 40 genetically distinct subtypes already identified, emphasizing the involvement of both the cerebellum and spinal cord, especially in SCAs with a CAG repeat expansion that encodes polyglutamine (polyQ) [4]. However, in some non-polyQ SCAs, the spinal cord is not primarily involved and the disease may arise in different anatomical structures, such as the basal ganglia or in peripheral nerves [5]. Moreover, several autosomal recessive or X-linked ataxias are also referred to as SCAs, with the addition of an R or X, respectively [6,7].

Other genetic forms of ataxia include dentatorubral-pallidoluysian atrophy [8], episodic ataxias (from 1 to 7) [9], Friedreich ataxia [10], ataxia-telangiectasia [11], ataxia with oculomotor apraxia (types 1 and 2) [12,13], Wilson’s disease [14], aceruloplasminemia [15], hereditary vitamin E deficiency [16], cerebellar ataxia neuropathy and vestibular areflexia syndrome (CANVAS) [17], cerebrotendinous xanthomatosis [18], fragile X-associated tremor/ataxia syndrome [19], and mitochondrial ataxias [20].

The most frequent acquired degenerative ataxias include the cerebellar variant of multiple system atrophy (MSA-C) [21,22] and, in some presentations, progressive supranuclear palsy (PSP) [23,24].

Currently, the majority of degenerative ataxias lack effective pharmacologic disease-modifying therapies and there is growing interest in finding innovative therapeutic approaches to improve clinical symptoms in patients with this spectrum of debilitating disorders. Recently, a report of the Guideline Development, Dissemination, and Implementation Subcommittee of the American Academy of Neurology has systematically reviewed the evidence regarding ataxia treatment, with only few studies emerging as promising for the treatment of only a subset of cerebellar ataxias [25]. What emerges is that the development of effective therapies may be hampered by the heterogeneity of the cerebellar ataxias and that specific therapeutic approaches may be required for each disease.

In this view, the field of non-invasive brain stimulation has recently gained much attention in the scientific community, in particular because stimulation techniques are non-invasive, provide novel information on cerebellar physiology, may modulate neural plasticity irrespectively of the underlying disease [26,27,28,29,30,31,32,33], and can be tailored to the needs of specific individuals patients.

In this review, we focus on the principal studies implementing non-invasive brain stimulation techniques, particularly transcranial magnetic stimulation (TMS) and transcranial direct current stimulation (tDCS), in the treatment of patients with degenerative ataxias.

We searched articles in English published between Jan 1, 1996 and Jan 31, 2020 on Medline (PubMed) using the terms “ataxia”, in combination with “transcranial direct current stimulation” OR “transcranial magnetic stimulation” OR “repetitive transcranial magnetic stimulation” OR “brain stimulation” OR “cerebellar stimulation”.

## 2. Transcranial Magnetic Stimulation for the Treatment of Cerebellar Ataxias

### 2.1. TMS Techniques

TMS is a noninvasive form of brain stimulation that uses electromagnetic induction as a highly effective, practically painless way to generate currents in the brain capable of discharging targeted neuronal populations. Magnetic fields readily penetrate into the brain without attenuation by the scalp or skull and generate a current according to Faraday’s law of electromagnetic induction [34].

Several TMS protocols, as single or paired-pulse stimulation techniques, have been used to assess the involvement of the corticospinal tract and motor cortex circuitry in hereditary cerebellar ataxias (for a review, see [35]). These studies have provided the basis for establishing objective biomarkers for early diagnosis and disease monitoring. Among them, the cerebellar inhibition protocol (CBI) has been shown to be particularly useful as a physiologic biomarker, as it appears to correlate with the improvement observed in clinical outcome measures [36,37,38,39]. The CBI protocol assesses the physiological inhibitory tone that the cerebellum exerts onto the primary motor cortex (M1), and has been shown to be impaired in cerebellar ataxias [36,40,41,42,43,44,45,46].

Contrary to single-pulse TMS, repetitive TMS (rTMS) is able to change and modulate cortical activity beyond the stimulation period as a potential method for treatment [47]. rTMS can be applied at various stimulation frequencies or as a patterned train of pulses and can be used in a variety of ways both in motor and non-motor brain regions and with local and nonlocal effects on brain activity [34]. In most individuals and most conditions, stimulation frequencies below 1 Hz result in suppression of activity in the targeted brain region, whereas repetition rates above 5 Hz are predominantly facilitatory [47,48]. However, substantial inter-individual variability has been well documented [49,50]. Moreover, patterned protocols such as theta burst stimulation (TBS) have been developed to induce a longer-lasting modulation of cortical excitability with a lower number of stimuli overall. There are two main protocols of TBS: intermittent TBS (iTBS) and continuous TBS (cTBS), which induce after-effects that resemble long-term potentiation (LTP) and long-term depression (LTD), respectively [51,52].

However, it should be noted that the mechanism of generating cerebellar plasticity may be very different from that observed in the motor cortex. NMDA-dependent LTP and LTD-like plasticity changes are based on experiments on hippocampal slices [52] and the mechanisms may be different from those in the cerebellar cortex. Indeed, the stimulation of parallel fibers at 1 Hz, which are likely to be the stimulated target of TMS, induces NMDAR-independent LTP [53]. Simultaneous activation of climbing fibers, or the Purkinje cell itself, as well as parallel fibers, actually leads to LTD, where a similar protocol in the cortex (by paired associated stimulation) typically causes LTP [53]. In general, the direction of plasticity changes by cerebellar stimulation could be very different, or even opposite to the classical view of NMDAR-dependent LTP applicable to the cerebral cortex.

### 2.2. Clinical Studies

The effects of low frequency rTMS were first evaluated by Shimizu and colleagues in four patients with SCA (two with SCA 6, one with SCA 1, one with SCA 7). They applied 10 TMS pulses at 100% of maximum stimulator output with an interpulse interval of more than 5 s for 21 consecutive days on the cerebellum and observed a reduction of the time required for a 10 m walk and in the number of steps in tandem gait in all patients. They also observed a significant increase in blood flow in the both cerebellar hemispheres, putamen, and pons with single photon emission computed tomography (SPECT) [54].

In a subsequent double-blind, sham-controlled trial performed by the same group, 74 patients with sporadic or hereditary spinocerebellar degeneration underwent a similar protocol with 10 TMS pulses applied over both cerebellar hemispheres and the inion for 21 consecutive days. They observed a greater improvement in gait speed and standing capacities in the intervention group compared to the sham group, with an increase in mean regional brain blood flow in the cerebellum and pons in the intervention group. Although placebo or training effects were demonstrated in gait and standing capacities, the effects of active stimulation were far beyond those of sham stimulation. They also observed in some patients that if cerebellar rTMS was repeated one or twice a week, the improvement persisted for at least 6 months. On the contrary, patients receiving rTMS every 2 weeks quickly returned to baseline conditions [55].

By applying a similar protocol for 8 weeks in 20 patients with spinocerebellar degeneration (10 with olivopontocerebellar atrophy, 6 with cortical cerebellar atrophy, 4 with SCA 6), Ihara and colleagues extended these results by observing a decrease in oxidative stress biomarkers after rTMS in most patients, which correlated with the improvement in clinical scores. They observed a significant decrease in ascorbate free radical concentrations in the cerebrospinal fluid of patients after rTMS compared to baseline. While before rTMS ascorbate free radical concentrations were significantly higher in patients compared to healthy subjects, after rTMS concentrations did not differ significantly between the two groups [56]. Oxidative stress is thought to concur in the pathology of several ataxias [57,58], with in vitro studies showing a neuroprotective effect of rTMS against oxidative stressors [59].

The same protocol was applied by Farzan and co-workers in one patient with idiopathic late-onset cerebellar atrophy for 21 days. They observed an improvement in dysmetria and tremor in the finger to nose and finger-chase tests in standing postural control and gait, evaluated kinematically and electromyographically, which were maintained at 6-month follow-up. They also assessed changes in CBI, observing a significant increase in facilitation at interstimulus intervals of 5, 6, and 7 ms (between the cerebellar and opposite motor cortex stimuli) [60].

By applying a slightly different protocol in a patient with Minamata disease, Nakamura and colleagues observed an improvement in gait and tremor only after real TMS but not after sham stimulation. They applied rTMS with the coil centered 4 cm lateral to the right of the inion (three trains), with the coil centered on the inion (four trains), and with the coil centered 4 cm lateral to the left of the inion (three trains). Each train consisted of 5 Hz stimulation for 10 s with a 50 s inter-train interval [61].

Another single-subject observation was made in a patient with SCA 6 by applying TMS over both the motor cortex and cerebellum for 5 days a week for 2 weeks, with an identical course repeated after 2 weeks. A total of 20 single pulses at approximately 0.3 Hz were applied twice over the motor cortices (20 with current flowing counter-clockwise and 20 clockwise, holding the coil over Cz), followed by 10 single pulses at 0.5 Hz applied twice over the cerebellum (10 with current flowing counter-clockwise and 10 clockwise). They observed a marked improvement in diplopia, particularly after stimulation of the motor cortices, accompanied by an improvement in limb ataxia, evaluated with the International Cooperative Ataxia Rating Scale (ICARS) [62].

Very recently, Dang and co-workers applied a slightly different protocol in another patient with SCA 6. They delivered 1500 pulses over the inion in 1 s trains at 10 Hz, with a 10 s inter-train interval. Each session was applied daily for 5 days a week for 20 weeks. They observed a significant improvement in posture and gait, kinetic functions, and speech, whereas no improvement in oculomotor movements was identified [63].

All of these observations paved the way for a randomized, double-blind, sham-controlled trial performed in 20 patients with genetically confirmed SCAs (1 with SCA 1, 1 with SCA 2, 13 with SCA 3, 3 with SCA 6, 1 with SCA 8, 1 with SCA 14), which was recently published. Manor and colleagues applied a 4-week, 20-session rTMS intervention targeting the cerebellum, guided by individual brain anatomy using structural magnetic resonance imaging (MRI). Ten pulses were delivered in every region, for a total of 30 pulses in each session. rTMS, compared to sham, was associated with greater improvement in the Scale for the Assessment and Rating of Ataxia (SARA) scores, particularly within the “stance” sub-score. This functional change was accompanied by improvement in several objective metrics of postural sway during eyes-open and eyes-closed standing. rTMS did not influence performance in the nine-hole peg test, the timed up-and-go test, or gait kinematics [64].

In summary, 122 patients have been assessed in 8 published studies using rTMS (see Table 1). All patients tolerated the interventions without complications and all published studies report beneficial effects in several domains. Several shortcomings, however, emerge: (1) protocols were different, including different stimulation parameters and durations of the interventions; (2) sample sizes were small, often with case reports or case series; (3) few studies were sham controlled; (4) blinding was insufficiently assessed; (5) outcome measures and reported benefits were variable; and (6) publication bias also makes reliability of the reported results uncertain.

## 3. Transcranial Direct Current Stimulation for the Treatment of Cerebellar Ataxias

### 3.1. tDCS Techniques

tDCS is a form of non-invasive brain stimulation that modulates neuronal excitability in a polarity-specific manner by delivering prolonged (10–20 min) but weak (1–2 mA) currents to brain tissues via electrodes placed on the scalp [65]. The effects of tDCS are thought to be primarily modulatory, modifying the likelihood of neuronal discharge by shifting membrane polarity. More specifically, in most instances anodal tDCS depolarizes the neuron’s resting membrane potential and thereby enhances the rate of spontaneous neuronal firing and increases cortical excitability, whereas cathodal tDCS conversely decreases cortical excitability by shifting resting membrane potential toward hyperpolarization, reducing the neuronal firing rate [66]. These polarity-dependent changes in cortical excitability, observed on the motor cortex, are hypothesized to depend on neuroplasticity mechanisms (NMDA-dependent processes) similar to those underlying LTP and LTD [67]. Nevertheless, as similarly reported for TMS, the plasticity changes induced by anodal tDCS on the cerebellar cortex may be very different from that observed on the motor cortex. Indeed, depolarizing cerebral cortical neurons/dendrites facilitate NMDA-dependent cascade in the post-synaptic side and lead to LTP. However, depolarizing dendrites of the cerebellar Purkinje cells leads to post-synaptic silencing of t-type calcium channel, which suppresses the effect of climbing fiber inputs and suppress intrinsic LTD [68]. Although anodal tDCS leads to “increased excitability” in both cerebral and cerebellar cortices, the fundamental mechanism could be very different (enhanced LTP and suppressed LTD, respectively).

Similarly to what has been observed in the motor cortex, cerebellar tDCS has been shown to modulate cerebellar excitability in a polarity-specific manner, with anodal cerebellar tDCS increasing the excitability of the cerebellar cortex and thus CBI, whereas cathodal cerebellar tDCS decreased CBI [69]. These polarity-specific effects were confirmed in subsequent behavioral studies performed in healthy controls [70,71,72,73,74]. However, as is the case for rTMS, substantial inter- and intra-individual variability of the tDCS has been increasingly noted.

### 3.2. Clinical Studies

Initial studies evaluated the effects of cerebellar tDCS on neurophysiological measures in ataxic patients. Grimaldi and colleagues observed in nine patients with several types of acquired and degenerative ataxias (one immune-mediated ataxia, one paraneoplastic ataxia, one recessive SCA, three SCA, three idiopathic adult-onset ataxias) a decrease in long latency stretch reflexes after tDCS stimulation with the anode over the right cerebellar hemisphere and the cathode over the contralateral supraorbital area. In this study, short latency stretch responses and coordination tasks in the upper limbs were unaffected [75].

The same group subsequently evaluated the effects of cerebello-cerebro tDCS in two patients with SCA 2. They observed that, after 20 min of stimulation with the anode over the right cerebellar hemisphere, immediately followed by 20 min of stimulation with the anode over the contralateral motor cortex and the cathode over the contralateral supraorbital area, tremor and hypermetria decreased in both patients. These results were accompanied by an improvement in SARA scores [76].

Taking into account that the excitability of the motor cortex is depressed in patients with cerebellar disease [77], Pozzi and collaborators tried to increase motor cortex excitability by placing the anode over the primary motor cortex of the affected side and the cathode over the contralateral primary motor cortex. By applying five sessions of 2 mA stimulation for 20 min in three patients with cerebellar ataxia (two with idiopathic late-onset ataxia, one with SCA 6), they observed an improvement in gait, stance, and sitting at the SARA score [78].

Following these promising results, several randomized, double-blind, sham-controlled clinical trials were performed in larger groups of patients with degenerative ataxia. One of the first was a randomized, double-blind, sham-controlled, crossover study in 19 patients with ataxia (one with SCA 1, five with SCA 2, two with SCA 38, one with Friedreich’s ataxia, one with ataxia with oculomotor apraxia type 2, one with fragile-X-associated tremor/ataxia syndrome, six with MSA-C, two with idiopathic late-onset ataxia). By applying a single session of 2 mA tDCS with the anode over the cerebellum and the cathode over the right deltoid muscle for 20 min, researchers observed a significant improvement in SARA and ICARS scores (particularly in posture, gait, and limb coordination), at the nine-hole peg test and at the 8 m walking time compared to the sham stimulation. They also observed a significant negative correlation between the improvement in clinical scores and the impairment on activities of daily living. These results were also confirmed when only SCA and MSA-C patients were considered separately [79].

Considering that repeated sessions of stimulation could have cumulative effects on synaptic plasticity [80], the same group applied the same protocol over two consecutive weeks (Monday to Friday) in a randomized, double-blind, sham-controlled trial in 20 patients with various types of ataxia (five SCA 2, one SCA 14, two SCA 38, one with Friedreich’s ataxia, one with ataxia with oculomotor apraxia type 2, one with fragile-X-associated tremor/ataxia syndrome, four with MSA-C, five with idiopathic late-onset ataxia). They observed that a 2-week treatment of tDCS with the anode over the cerebellum and the cathode over the right deltoid muscle significantly improved all performance scores (SARA, ICARS, nine-hole peg test, 8 m walking time) and increased CBI compared to sham stimulation, which were persistent at 3 month follow-up. The improvement in clinical scores significantly correlated with the restoration of CBI. As in the previous study, there was a greater improvement in patients with a reduced impairment in functional scores at baseline [81].

Trying to increase the possible effects of tDCS in patients with ataxia, which frequently have an involvement of the spinal cord, the same group performed a randomized, double-blind, sham-controlled, crossover trial in 21 patients with neurodegenerative ataxia (seven SCA 2, one SCA 14, one SCA 38, one with Friedreich’s ataxia, one with ataxia with oculomotor apraxia type 2, six with MSA-C, four with idiopathic late-onset ataxia) by applying a concurrent stimulation with the anode over the cerebellum and the cathode over the spinal lumbar enlargement (2 cm under T11) for two consecutive weeks (Monday to Friday). Additionally in this case, cerebello-spinal tDCS showed a significant improvement in all performance scores (SARA, ICARS, nine-hole peg test, 8 m walking time), in motor cortex excitability, and CBI compared to sham stimulation [82,83].

A following double-blind, auto-matched clinical trial performed in seven patients with ataxia (four with slowly progressive cerebellar ataxia, three with non-progressive cerebellar ataxia) showed a significant improvement of gait parameters and SARA scores after five sessions of tDCS with the anode targeting both motor cortices (20 min on the left followed by 20 min on the right motor cortex) and the cathode over the contralateral supraorbital area [84].

An important step forward was achieved by Pilloni and co-workers, who evaluated the feasibility of long-term at-home treatment with cerebellar tDCS in a patient with cerebellar ataxia. They delivered 60 tDCS sessions, 59 of which were administered remotely, observing an improvement in gait speed and manual dexterity. The applied tDCS montage involved placing the anode over the cerebellum and the cathode over the right shoulder [85]. These findings lay the foundations for the future application of supervised at-home treatment for prolonged periods in patients with ataxia.

On the contrary, two studies performed by the same group did not observe any significant effects of cerebellar tDCS in patients with ataxia. Hulst and co-workers assessed in 20 patients with cerebellar degeneration (five SCA 6, three SCA 14, four with autosomal dominant cerebellar ataxia type III, seven with idiopathic adult-onset ataxia, one with cerebellar degeneration caused by cerebellitis) the effects of approximately 22 min of tDCS with the anode over the right cerebellar hemisphere and the cathode over the right buccinator muscle. After 1 week, patients received another session of tDCS with the anode over M1 and the cathode over the contralateral supraorbital region. They did not observe any significant effects after cerebellar or M1 stimulation in a standard reaching task with force-field perturbations [86]. By applying the same protocol, John and colleagues did not observe significant after-effects of tDCS on grip force control in 14 patients with cerebellar degeneration (two with SCA 6, three SCA 14, five with autosomal dominant cerebellar ataxia type III, three with idiopathic adult-onset ataxia, one with cerebellar degeneration caused by cerebellitis) [87]. Several factors could have influenced these negative outcomes. Stimulation parameters, such as electrode montage, number of sessions, timing of the onset of stimulation, the selection of tasks that may not entirely depend on cerebellar structures, study design with possible carryover effects between sessions, and sample size, are relevant factors that could explain some of these negative results [88], but further studies are needed clarify these aspects.

Another relevant aspect that should be more systematically assessed in tDCS studies is the effect of sham tDCS, which is fundamental due to the placebo response observed in non-invasive brain stimulation trials [89]. Sham-controlled tDCS studies have frequently yielded inconsistent results, which might arise in part from sham inconsistencies. Indeed, a multiplicity of sham stimulation protocols are being used in the tDCS research field and might have different biological effects beyond the intended transient sensations. Whether certain modalities of sham tDCS, and if the cumulative clinical effects of low-intensity, repeated sham tDCS, have a neuro-biological effect is still to be elucidated. More research is necessary to establish the direct neurobiological effects of sham tDCS protocols and evaluate their reliability [90,91].

In summary, 116 patients have been assessed in 10 published studies using tDCS (see Table 2). All patients tolerated the interventions without complications and most published studies report beneficial effects in several domains. Several shortcomings, however, emerge: (1) the majority of studies did not use modelling to fully capture the induced electric fields and did not optimize the “dose” for each participant; (2) protocols were different, including different electrode sizes, electrode shapes, electrode locations, intensity, and durations of the interventions; (3) sample sizes were small, often with case reports or case series; (4) few studies were sham controlled, and sham tDCS may be problematic [92,93]; (5) blinding was insufficiently assessed; and (6) outcome measures and reported benefits were variable.

## 4. Conclusions

Cerebellar ataxias are a very heterogenous group of degenerative disorders for which we currently lack effective and disease-modifying interventions. The field of non-invasive brain stimulation has made much progress in the development of specific stimulation protocols to modulate cerebellar excitability and try to restore the physiological activity of the cerebellum in patients with ataxia but also in other neurodegenerative disorders [31]. Several different stimulation protocols have emerged, with both rTMS and tDCS. A significant limitation of these studies is that methods frequently differ by a considerable degree regarding the area of stimulation, intensity, and number of sessions, including different clinical or neurophysiological outcome measures. Moreover, the relative infrequency of cerebellar ataxias limits the design of sufficiently powered and homogenous clinical trials. Recently, the rationale and protocol of a randomized, double-blind, sham-controlled clinical trial in patients with only SCA 3 has been published and is underway [94].

Nevertheless, several studies both in healthy controls and in patients with ataxia have suggested that the modulation of the cerebellum by non-invasive brain stimulation may enhance postural control, gait, and motor coordination. Although the effects of cerebellar stimulation on cognitive functions have been abundantly studied in healthy controls [27], there is a lack of studies assessing the effect on cognition an emotions in patients with cerebellar ataxia. It is now clear that degenerative ataxias frequently have an impairment in several cognitive domains [95], which have been encompassed in the cerebellar cognitive affective syndrome, characterized by deficits in executive functions, linguistic processing, spatial cognition, and affect regulation [96,97].

Despite the promising findings, the precise mechanism of action of non-invasive cerebellar stimulation still remains largely unknown. Other than modulating the excitability of Purkinje cells and increasing their activity on the dentato-thalamo-cortical pathway, several other mechanisms could be involved, such as inactivation or activation of specific cellular processes, including gene expression, protein synthesis, channel pump regulation, and receptor or neurotransmitter modulation [27].

Several questions remain unanswered and could provide novel targets for future studies. For example, several studies have now shown that the effects of iTBS might be even more relevant than those obtained after rTMS [98], and encouraging results have been achieved in the treatment of gait and balance recovery in patients with stroke after cerebellar iTBS [99,100]. Moreover, transcranial alternating current stimulation (tACS) also has recently emerged as a new technique to modulate cortical oscillations and entrain brain rhythms in specific frequencies [101]. By applying cerebellar tACS at a frequency matching the basal firing rate of Purkinje cells (50 Hz), researchers have shown that tACS may modulate CBI and improve the performance of a motor tasks in healthy controls [102,103,104,105,106]. Whether these novel types of stimulation might be effective also in the treatment of cerebellar ataxias is still unknown.

Future studies should try to assess several issues: the inclusion of etiologically homogenous group of patients, perhaps in multicenter studies; defining the optimal timing of follow-up stimulation sessions and the effects of repeated sessions over time; the effects on cognition and emotions; the feasibility of at-home remotely supervised stimulation in larger cohorts; and if these effects may be intensified by concurrent motor training interventions or pharmacologic therapies. Critically, evaluation of target engagement using imaging or physiologic biomarkers, as well as the assessment of “dose” by using modelling to calculate the induced currents in the brain to define individual stimulation parameters, would be essential. Moreover, the cerebellum can show significant structural changes in term of climbing fiber Purkinje cell and parallel fiber Purkinje cell wirings in cerebellar ataxia and other disorders [102,103,104,105,106], and some wiring features can be rapidly changed within days and affect cerebellar synchronization [107]. These structural changes may affect the effect of TMS and tDCS, and should be considered future studies.

In conclusion, particularly in light of limited evidence-based pharmacologic and non-pharmacologic treatment options for patients with ataxia, non-invasive brain stimulation techniques with rTMS or tDCS are potentially promising tools for therapeutic approaches, but further work is needed before it can be broadly offered in the clinical setting.

## Figures and Tables

**Table 1:** Studies assessing the effects of repetitive transcranial magnetic stimulation (rTMS) in patients with cerebellar ataxia.

Study	Patients	Sham	Blinding	Stimulation	Protocol
[54]	4	No	Not reported	Inion and cerebellar hemispheres	30 pulses (100% MSO) at 0.17 Hz every day for 21 days
[55]	74	Yes	Patients and examiners	Inion and cerebellar hemispheres	30 pulses (100% MSO) at 0.17 Hz every day for 21 days
[56]	20	No	Yes	Inion and cerebellar hemispheres	30 pulses (100% MSO) at 0.2 Hz every day for 8 weeks
[60]	1	No	Not reported	Inion and cerebellar hemispheres	30 pulses (100% MSO) at 0.17 Hz every day for 21 days
[61]	1	Yes	Not reported	Inion and cerebellar hemispheres	500 pulses (90% RMT) at 5 Hz for 10 s with a 50 s interval, every day for 2 days/week for 4 months
[62]	1	Yes	Not reported	Motor cortices and cerebellar hemispheres	40 pulses (100% RMT) over Cz at 0.2 Hz + 20 pulses (50% RMT) over inion at 0.5 Hz every day for 4 weeks
[63]	1	No	Not reported	Inion	1500 pulses (100% MSO) at 10 Hz for 1 s with a 10 s interval, every day for 4 weeks
[64]	20	Yes	Yes	Inion and cerebellar hemispheres	30 pulses (100% MSO) at 0.17 Hz every day for 21 days

MSO: maximum stimulator output; RMT: resting motor threshold.

**Table 2:** Studies assessing the effects of transcranial direct current stimulation (tDCS) in patients with cerebellar ataxia.

Study	Patients	Sham	Blinding	Anode	Cathode	Protocol
[75]	9	Yes	Patients	Right cerebellar hemisphere	L supraorbital area	1–2 mA, 20 min
[76]	2	Yes	Patients	Right cerebellar hemisphere/left motor cortex	Contralateral supraorbital area	1 mA, 20 min
[78]	3	Yes	Patients and examiners	Motor cortex affected side	Motor cortex unaffected side	2 mA, 20 min for five sessions
[79]	19	Yes	Patients and examiners	Cerebellar hemispheres	Right deltoid muscle	2 mA, 20 min
[81]	20	Yes	Patients and examiners	Cerebellar hemispheres	Right deltoid muscle	2 mA, 20 min for 10 days
[82]	21	Yes	Patients and examiners	Cerebellar hemispheres	Spinal lumbar enlargement	2 mA, 20 min for 10 days
[84]	7	Yes	Patients and examiners	Motor cortices	Contralateral supraorbital area	2 mA, 20 min for five days
[85]	1	No	Not reported	Cerebellar hemispheres	Right shoulder	2.5 mA, 20 min for 60 days
[86]	20	Yes	Patients and examiners	Right cerebellar hemisphere/motor cortex	Right buccinator muscle/contralateral supraorbital region	2 mA, 22 min
[87]	14	Yes	Patients and examiners	Right cerebellar hemisphere/motor cortex	Right buccinator muscle/contralateral supraorbital region	2 mA, 22 min

## Abbreviations

CANVAS	cerebellar ataxia neuropathy and vestibular areflexia syndrome
CBI	cerebellar brain inhibition
ICARS	International Cooperative Ataxia Rating Scale
LTD	long-term depression
LTP	long-term potentiation
MSCA-C	cerebellar variant of multiple system atrophy
PSP	progressive supranuclear palsy
SARA	Scale for the Assessment and Rating of Ataxia
SCA	spinocerebellar ataxia
tACS	transcranial alternating current stimulation
tDCS	transcranial direct current stimulation
TBS	theta burst stimulation
TMS	transcranial magnetic stimulation
rTMS	repetitive transcranial magnetic stimulation

## References

[1] Harding A.E. (1983). Classification of the Hereditary Ataxias and Paraplegias. Lancet.

[2] Durr A. (2010). Autosomal dominant cerebellar ataxias: Polyglutamine expansions and beyond. Lancet Neurol..

[3] Ruano L., Melo C., Silva M.C., Coutinho P. (2014). The Global Epidemiology of Hereditary Ataxia and Spastic Paraplegia: A Systematic Review of Prevalence Studies. Neuroepidemiology.

[4] Paulson H.L., Shakkottai V.G., Clark H.B., Orr H.T. (2017). Polyglutamine spinocerebellar ataxias — From genes to potential treatments. Nat. Rev. Neurosci..

[5] Klockgether T., Mariotti C., Paulson H.L. (2019). Spinocerebellar ataxia. Nat. Rev. Dis. Prim..

[6] Synofzik M., Németh A.H. (2018). Recessive ataxias. Handb. Clin. Neurol..

[7] Zanni G., Bertini E., Manto M., Huisman T.A.G.M. (2018). X-linked ataxias. Handbook of Clinical Neurology.

[8] Nagafuchi S., Yanagisawa H., Ohsaki E., Shirayama T., Tadokoro K., Inoue T., Yamada M. (1994). Structure and expression of the gene responsible for the triplet repeat disorder, dentatorubral and pallidoluysian atrophy (DRPLA). Nat. Genet..

[9] Jen J.C., Graves T.D., Hess E.J., Hanna M.G., Griggs R.C., Baloh R.W. (2007). Primary episodic ataxias: Diagnosis, pathogenesis and treatment. Brain.

[10] Dürr A., Cossee M., Agid Y., Campuzano V., Mignard C., Penet C., Mandel J.L., Brice A., Koenig M. (1996). Clinical and genetic abnormalities in patients with Friedreich’s ataxia. N. Engl. J. Med..

[11] Gatti R.A., Berkel I., Boder E., Braedt G., Charmley P., Concannon P., Ersoy F., Foroud T., Jaspers N.G., Lange K. (1988). Localization of an ataxia-telangiectasia gene to chromosome 11q22–23. Nature.

[12] Le Ber I., Moreira M.C., Rivaud-Péchoux S., Chamayou C., Ochsner F., Kuntzer T., Tardieu M., Saïd G., Habert M.O., Demarquay G. (2003). Cerebellar ataxia with oculomotor apraxia type 1: Clinical and genetic studies. Brain.

[13] Le Ber I., Bouslam N., Rivaud-Péchoux S., Guimarães J., Benomar A., Chamayou C., Goizet C., Moreira M.C., Klur S., Yahyaoui M. (2004). Frequency and phenotypic spectrum of ataxia with oculomotor apraxia 2: A clinical and genetic study in 18 patients. Brain.

[14] Mai N., Bolsinger P., Avarello M., Diener H.C., Dichgans J. (1988). Control of isometric finger force in patients with cerebellar disease. Brain.

[15] Roy C.N., Andrews N.C. (2001). Recent advances in disorders of iron metabolism: Mutations, mechanisms and modifiers. Hum. Mol. Genet..

[16] Ouahchi K., Arita M., Kayden H., Hentati F., Hamida M.B., Sokol R., Arai H., Inoue K., Mandel J.L., Koenig M. (1995). Ataxia with isolated vitamin E deficiency is caused by mutations in the α–tocopherol transfer protein. Nat. Genet..

[17] Cortese A., Simone R., Sullivan R., Vandrovcova J., Tariq H., Yan Y.W., Humphrey J., Jaunmuktane Z., Sivakumar P., Polke J. (2019). Biallelic expansion of an intronic repeat in RFC1 is a common cause of late-onset ataxia. Nat. Genet..

[18] Cali J.J., Hsieh C.L., Francke U., Russell D.W. (1991). Mutations in the bile acid biosynthetic enzyme sterol 27-hydroxylase underlie cerebrotendinous xanthomatosis. J. Biol. Chem..

[19] Hagerman R., Hagerman P. (2013). Advances in clinical and molecular understanding of the FMR1 premutation and fragile X-associated tremor/ataxia syndrome. Lancet Neurol..

[20] Zeviani M., Simonati A., Bindoff L.A., Subramony S.H., Dürr A. (2012). Ataxia in mitochondrial disorders. Handbook of Clinical Neurology.

[21] Gilman S., Wenning G.K., Low P.A., Brooks D.J., Mathias C.J., Trojanowski J.Q., Wood N.W., Colosimo C., Dürr A., Fowler C.J. (2008). Second consensus statement on the diagnosis of multiple system atrophy. Neurology.

[22] Schwartz S., Besenthal I., Dichgans J., Zu C., Scho L., Riess O., Abele M., Bu K. (2002). The aetiology of sporadic adult-onset ataxia. Brain.

[23] Kanazawa M., Shimohata T., Toyoshima Y., Tada M., Kakita A., Morita T., Ozawa T., Takahashi H., Nishizawa M. (2009). Cerebellar involvement in progressive supranuclear palsy: A clinicopathological study. Mov. Disord..

[24] Höglinger G.U., Respondek G., Stamelou M., Kurz C., Josephs K.A., Lang A.E., Mollenhauer B., Müller U., Nilsson C., Whitwell J.L. (2017). Clinical diagnosis of progressive supranuclear palsy: The movement disorder society criteria. Mov. Disord..

[25] Zesiewicz T.A., Wilmot G., Kuo S.-H., Perlman S., Greenstein P.E., Ying S.H., Ashizawa T., Subramony S.H., Schmahmann J.D., Figueroa K.P. (2018). Comprehensive systematic review summary: Treatment of cerebellar motor dysfunction and ataxia. Neurology.

[26] Maas R.P.P.W.M., Helmich R.C.G., van de Warrenburg B.P.C. (2019). The role of the cerebellum in degenerative ataxias and essential tremor: Insights from noninvasive modulation of cerebellar activity. Mov. Disord..

[27] Grimaldi G., Argyropoulos G.P., Bastian A., Cortes M., Davis N.J., Edwards D.J., Ferrucci R., Fregni F., Galea J.M., Hamada M. (2016). Cerebellar Transcranial Direct Current Stimulation (ctDCS): A Novel Approach to Understanding Cerebellar Function in Health and Disease. Neuroscientist.

[28] Manto M., Taib N.O. (2008). Ben A novel approach for treating cerebellar ataxias. Med. Hypotheses.

[29] Grimaldi G., Argyropoulos G.P., Boehringer A., Celnik P., Edwards M.J., Ferrucci R., Galea J.M., Groiss S.J., Hiraoka K., Kassavetis P. (2014). Non-invasive cerebellar stimulation—A consensus paper. Cerebellum.

[30] Gandini J., Manto M., Bremova-Ertl T., Feil K., Strupp M. (2020). The neurological update: Therapies for cerebellar ataxias in 2020. J. Neurol..

[31] Ferrucci R., Bocci T., Cortese F., Ruggiero F., Priori A. (2016). Cerebellar transcranial direct current stimulation in neurological disease. Cerebellum Ataxias.

[32] Mitoma H., Manto M. (2018). The Era of Cerebellar Therapy. Curr. Neuropharmacol..

[33] Miterko L.N., Baker K.B., Beckinghausen J., Bradnam L.V., Cheng M.Y., Cooperrider J., DeLong M.R., Gornati S.V., Hallett M., Heck D.H. (2019). Consensus Paper: Experimental Neurostimulation of the Cerebellum. Cerebellum.

[34] Rossini P.M., Burke D., Chen R., Cohen L.G., Daskalakis Z., Di Iorio R., Di Lazzaro V., Ferreri F., Fitzgerald P.B., George M.S. (2015). Non-invasive electrical and magnetic stimulation of the brain, spinal cord, roots and peripheral nerves: Basic principles and procedures for routine clinical and research application. An updated report from an I.F.C.N. Committee. Clin. Neurophysiol..

[35] Rodríguez-Labrada R., Velázquez-Pérez L., Ziemann U. (2018). Transcranial magnetic stimulation in hereditary ataxias: Diagnostic utility, pathophysiological insight and treatment. Clin. Neurophysiol..

[36] Ugawa Y., Terao Y., Nagai C., Nakamura K., Kanazawa I. (1995). Electrical stimulation of the cerebellum normally suppresses motor cortical excitability in a patient with ataxia due to a lesion of the middle cerebellar peduncle. Eur. Neurol..

[37] Ugawa Y., Uesaka Y., Terao Y., Hanajima R., Kanazawa I. (1995). Magnetic stimulation over the cerebellum in humans. Ann. Neurol..

[38] Benussi A., Dell’Era V., Cantoni V., Turrone R., Pilotto A., Alberici A., Cotelli M.S., Rizzetti C., Padovani A., Borroni B. (2019). Stimulation over the cerebellum with a regular figure-of-eight coil induces reduced motor cortex inhibition in patients with progressive supranuclear palsy. Brain Stimul..

[39] Fernandez L., Major B.P., Teo W.-P., Byrne L.K., Enticott P.G. (2018). Assessing cerebellar brain inhibition (CBI) via transcranial magnetic stimulation (TMS): A systematic review. Neurosci. Biobehav. Rev..

[40] Ugawa Y., Terao Y., Hanajima R., Sakai K., Furubayashi T., Machii K., Kanazawa I. (1997). Magnetic stimulation over the cerebellum in patients with ataxia. Electroencephalogr. Clin. Neurophysiol..

[41] Ugawa Y., Genba K., Iwata M., Kanazawa I. (1993). Suppression of motor cortical excitability by cerebellar stimulation in ataxia. Electroencephalogr. Clin. Neurophysiol..

[42] Matsunaga K., Uozumi T., Hashimoto T., Tsuji S. (2001). Cerebellar stimulation in acute cerebellar ataxia. Clin. Neurophysiol..

[43] Ugawa Y., Genba-Shimizu K., Rothwell J.C., Iwata M., Kanazawa I. (1994). Suppression of motor cortical excitability by electrical stimulation over the cerebellum in ataxia. Ann. Neurol..

[44] Ugawa Y., Hanajima R., Kanazawa I. (1994). Motor cortex inhibition in patients with ataxia. Electroencephalogr. Clin. Neurophysiol..

[45] Iwata N., Ugawa Y. (2005). The effects of cerebellar stimulation on the motor cortical excitability in neurological disorders: A review. Cerebellum.

[46] Matsugi A., Kikuchi Y., Kaneko K., Seko Y., Odagaki M. (2018). Cerebellar transcranial magnetic stimulation facilitates excitability of spinal reflex, but does not affect cerebellar inhibition and facilitation in spinocerebellar ataxia. Neuroreport.

[47] Pascual-Leone A., Valls-Solé J., Wassermann E.M., Hallett M. (1994). Responses to rapid-rate transcranial magnetic stimulation of the human motor cortex. Brain.

[48] Maeda F., Keenan J.P., Tormos J.M., Topka H., Pascual-Leone A. (2000). Modulation of corticospinal excitability by repetitive transcranial magnetic stimulation. Clin. Neurophysiol..

[49] Davila-Pérez P., Jannati A., Fried P.J., Cudeiro Mazaira J., Pascual-Leone A. (2018). The Effects of Waveform and Current Direction on the Efficacy and Test–Retest Reliability of Transcranial Magnetic Stimulation. Neuroscience.

[50] Maeda F., Keenan J.P., Tormos J.M., Topka H., Pascual-Leone A. (2000). Interindividual variability of the modulatory effects of repetitive transcranial magnetic stimulation on cortical excitability. Exp. Brain Res..

[51] Huang Y.-Z., Edwards M.J., Rounis E., Bhatia K.P., Rothwell J.C. (2005). Theta burst stimulation of the human motor cortex. Neuron.

[52] Bliss T.V.P., Lømo T. (1973). Long-lasting potentiation of synaptic transmission in the dentate area of the anaesthetized rabbit following stimulation of the perforant path. J. Physiol..

[53] Wang D.J., Su L.D., Wang Y.N., Yang D., Sun C.L., Zhou L., Wang X.X., Shen Y. (2014). Long-term potentiation at cerebellar parallel fiber-Purkinje cell synapses requires presynaptic and postsynaptic signaling cascades. J. Neurosci..

[54] Shimizu H., Tsuda T., Shiga Y., Miyazawa K., Onodera Y., Matsuzaki M., Nakashima I., Furukawa K., Aoki M., Kato H. (1999). Therapeutic efficacy of transcranial magnetic stimulation for hereditary spinocerebellar degeneration. Tohoku J. Exp. Med..

[55] Shiga Y., Tsuda T., Itoyama Y., Shimizu H., Miyazawa K.-I., Jin K., Yamazaki T. (2002). Transcranial magnetic stimulation alleviates truncal ataxia in spinocerebellar degeneration. J. Neurol. Neurosurg. Psychiatry.

[56] Ihara Y., Nobukuni K., Takata H., Sakai K., Nishinaka T., Tanabe Y., Takahashi K. (2006). Influence of repetitive transcranial magnetic stimulation on ataxia severity and biochemical parameters in cerebrospinal fluid of patients with spinocerebellar degeneration. IRYO Jpn. J. Natl. Med. Serv..

[57] Schulz J.B., Dehmer T., Schöls L., Mende H., Hardt C., Vorgerd M., Bürk K., Matson W., Dichgans J., Beal M.F. (2000). Oxidative stress in patients with Friedreich ataxia. Neurology.

[58] Yamashita T., Ando Y., Obayashi K., Terazaki H., Sakashita N., Uchida K., Ohama E., Ando M., Uchino M. (2000). Oxidative injury is present in Purkinje cells in patients with olivopontocerebellar atrophy. J. Neurol. Sci..

[59] Post A., Müller M.B., Engelmann M., Keck M.E. (1999). Repetitive transcranial magnetic stimulation in rats: Evidence for a neuroprotective effect in vitro and in vivo. Eur. J. Neurosci..

[60] Farzan F., Wu Y., Manor B., Anastasio E.M., Lough M., Novak V., Greenstein P.E., Pascual-Leone A. (2013). Cerebellar TMS in treatment of a patient with cerebellar ataxia: Evidence from clinical, biomechanics and neurophysiological assessments. Cerebellum.

[61] Nakamura M., Bekki M., Miura Y., Itatani M., Jie L.X. (2019). Cerebellar Transcranial Magnetic Stimulation Improves Ataxia in Minamata Disease. Case Rep. Neurol..

[62] Kawamura K., Etoh S., Shimodozono M. (2018). Transcranial magnetic stimulation for diplopia in a patient with spinocerebellar ataxia type 6: A case report. Cerebellum Ataxias.

[63] Dang G., Su X., Zhou Z., Che S., Zeng S., Chen S., Guo Y. (2019). Beneficial effects of cerebellar rTMS stimulation on a patient with spinocerebellar ataxia type 6. Brain Stimul..

[64] Manor B., Greenstein P.E., Davila-Perez P., Wakefield S., Zhou J., Pascual-Leone A. (2019). Repetitive transcranial magnetic stimulation in spinocerebellar ataxia: A pilot randomized controlled trial. Front. Neurol..

[65] Pellicciari M.C., Miniussi C. (2018). Transcranial Direct Current Stimulation in Neurodegenerative Disorders. J. ECT.

[66] Nitsche M.A., Paulus W. (2000). Excitability changes induced in the human motor cortex by weak transcranial direct current stimulation. J. Physiol..

[67] Liebetanz D., Nitsche M.A., Tergau F., Paulus W. (2002). Pharmacological approach to the mechanisms of transcranial DC-stimulation-induced after-effects of human motor cortex excitability. Brain.

[68] Hoxha E., Balbo I., Miniaci M.C., Tempia F. (2018). Purkinje cell signaling deficits in animal models of ataxia. Front. Synaptic Neurosci..

[69] Galea J.M., Jayaram G., Ajagbe L., Celnik P. (2009). Modulation of Cerebellar Excitability by Polarity-Specific Noninvasive Direct Current Stimulation. J. Neurosci..

[70] Jayaram G., Tang B., Pallegadda R., Vasudevan E.V.L., Celnik P., Bastian A. (2012). Modulating locomotor adaptation with cerebellar stimulation. J. Neurophysiol..

[71] Galea J.M., Vazquez A., Pasricha N., Orban de Xivry J.J., Celnik P. (2011). Dissociating the Roles of the Cerebellum and Motor Cortex during Adaptive Learning: The Motor Cortex Retains What the Cerebellum Learns. Cereb. Cortex.

[72] Poortvliet P., Hsieh B., Cresswell A., Au J., Meinzer M. (2018). Cerebellar transcranial direct current stimulation improves adaptive postural control. Clin. Neurophysiol..

[73] Cantarero G., Spampinato D., Reis J., Ajagbe L., Thompson T., Kulkarni K., Celnik P. (2015). Cerebellar Direct Current Stimulation Enhances On-Line Motor Skill Acquisition through an Effect on Accuracy. J. Neurosci..

[74] Batsikadze G., Rezaee Z., Chang D.I., Gerwig M., Herlitze S., Dutta A., Nitsche M.A., Timmann D. (2019). Effects of cerebellar transcranial direct current stimulation on cerebellar-brain inhibition in humans: A systematic evaluation. Brain Stimul..

[75] Grimaldi G., Manto M. (2013). Anodal transcranial direct current stimulation (tDCS) decreases the amplitudes of long-latency stretch reflexes in cerebellar ataxia. Ann. Biomed. Eng..

[76] Grimaldi G., Oulad Ben Taib N., Manto M., Bodranghien F. (2014). Marked reduction of cerebellar deficits in upper limbs following transcranial cerebello-cerebral DC stimulation: Tremor reduction and re-programming of the timing of antagonist commands. Front. Syst. Neurosci..

[77] Hore J., Flament D. (1988). Changes in motor cortex neural discharge associated with the development of cerebellar limb ataxia. J. Neurophysiol..

[78] Pozzi N.G., Minafra B., Zangaglia R., Marzi R., Sandrini G., Priori A., Pacchetti C. (2013). Transcranial Direct Current Stimulation (tDCS) of the Cortical Motor Areas in Three Cases of Cerebellar Ataxia. Cerebellum.

[79] Benussi A., Koch G., Cotelli M., Padovani A., Borroni B. (2015). Cerebellar transcranial direct current stimulation in patients with ataxia: A double-blind, randomized, sham-controlled study. Mov. Disord..

[80] Brunoni A.R., Nitsche M.A., Bolognini N., Bikson M., Wagner T., Merabet L., Edwards D.J., Valero-Cabre A., Rotenberg A., Pascual-Leone A. (2012). Clinical research with transcranial direct current stimulation (tDCS): Challenges and future directions. Brain Stimul..

[81] Benussi A., Dell’Era V., Cotelli M.S., Turla M., Casali C., Padovani A., Borroni B. (2017). Long term clinical and neurophysiological effects of cerebellar transcranial direct current stimulation in patients with neurodegenerative ataxia. Brain Stimul..

[82] Benussi A., Dell’Era V., Cantoni V., Bonetta E., Grasso R., Manenti R., Cotelli M., Padovani A., Borroni B. (2018). Cerebello-spinal tDCS in ataxia: A randomized, double-blind, sham-controlled, crossover trial. Neurology.

[83] Benussi A., Borroni B. (2019). Author response: Cerebello-spinal tDCS in ataxia: A randomized, double-blind, sham-controlled, crossover trial. Neurology.

[84] Barretto T.L., Bandeira I.D., Jagersbacher J.G., Barretto B.L., de Oliveira e Torres Â.F.S., Peña N., Miranda J.G.V., Lucena R. (2019). Transcranial direct current stimulation in the treatment of cerebellar ataxia: A two-phase, double-blind, auto-matched, pilot study. Clin. Neurol. Neurosurg..

[85] Pilloni G., Shaw M., Feinberg C., Clayton A., Palmeri M., Datta A., Charvet L.E. (2019). Long term at-home treatment with transcranial direct current stimulation (tDCS) improves symptoms of cerebellar ataxia: A case report. J. Neuroeng. Rehabil..

[86] Hulst T., John L., Küper M., Van Der Geest J.N., Göricke S.L., Donchin O., Timmann D. (2017). Cerebellar patients do not benefit from cerebellar or M1 transcranial direct current stimulation during force-field reaching adaptation. J. Neurophysiol..

[87] John L., Küper M., Hulst T., Timmann D., Hermsdörfer J. (2017). Effects of transcranial direct current stimulation on grip force control in patients with cerebellar degeneration. Cerebellum Ataxias.

[88] Biabani M., Aminitehrani M., Zoghi M., Farrell M., Egan G., Jaberzadeh S. (2017). The effects of transcranial direct current stimulation on short-interval intracortical inhibition and intracortical facilitation: A systematic review and meta-analysis. Rev. Neurosci..

[89] Razza L.B., Moffa A.H., Moreno M.L., Carvalho A.F., Padberg F., Fregni F., Brunoni A.R. (2018). A systematic review and meta-analysis on placebo response to repetitive transcranial magnetic stimulation for depression trials. Prog. Neuro-Psychopharmacol. Biol. Psychiatry.

[90] Horvath J.C., Forte J.D., Carter O. (2015). Evidence that transcranial direct current stimulation (tDCS) generates little-to-no reliable neurophysiologic effect beyond MEP amplitude modulation in healthy human subjects: A systematic review. Neuropsychologia.

[91] Antal A., Keeser D., Priori A., Padberg F., Nitsche M.A. (2015). Conceptual and procedural shortcomings of the systematic review “evidence that transcranial direct current stimulation (tDCS) generates little-to-no reliable neurophysiologic effect beyond MEP amplitude modulation in healthy human subjects: A systematic review” by horvath and co-workers. Brain Stimul..

[92] Fonteneau C., Mondino M., Arns M., Baeken C., Bikson M., Brunoni A.R., Burke M.J., Neuvonen T., Padberg F., Pascual-Leone A. (2019). Sham tDCS: A hidden source of variability? Reflections for further blinded, controlled trials. Brain Stimul..

[93] Neri F., Mencarelli L., Menardi A., Giovannelli F., Rossi S., Sprugnoli G., Rossi A., Pascual-Leone A., Salvador R., Ruffini G. (2020). A novel tDCS sham approach based on model-driven controlled shunting. Brain Stimul..

[94] Maas R.P.P.W.M., Toni I., Doorduin J., Klockgether T., Schutter D.J.L.G., Van De Warrenburg B.P.C. (2019). Cerebellar transcranial direct current stimulation in spinocerebellar ataxia type 3 (SCA3-tDCS): Rationale and protocol of a randomized, double-blind, sham-controlled study. BMC Neurol..

[95] Teive H.A.G., Arruda W.O. (2009). Cognitive dysfunction in spinocerebellar ataxias. Dement. Neuropsychol..

[96] Hoche F., Guell X., Vangel M.G., Sherman J.C., Schmahmann J.D. (2018). The cerebellar cognitive affective/Schmahmann syndrome scale. Brain.

[97] Schmahmann J.D., Sherman J.C. (1998). The cerebellar cognitive affective syndrome. Brain.

[98] Blumberger D.M., Vila-Rodriguez F., Thorpe K.E., Feffer K., Noda Y., Giacobbe P., Knyahnytska Y., Kennedy S.H., Lam R.W., Daskalakis Z.J. (2018). Effectiveness of theta burst versus high-frequency repetitive transcranial magnetic stimulation in patients with depression (THREE-D): A randomised non-inferiority trial. Lancet.

[99] Koch G., Bonnì S., Casula E.P., Iosa M., Paolucci S., Pellicciari M.C., Cinnera A.M., Ponzo V., Maiella M., Picazio S. (2019). Effect of Cerebellar Stimulation on Gait and Balance Recovery in Patients With Hemiparetic Stroke: A Randomized Clinical Trial. JAMA Neurol..

[100] Bonnì S., Ponzo V., Caltagirone C., Koch G. (2014). Cerebellar theta burst stimulation in stroke patients with ataxia. Funct. Neurol..

[101] Antal A., Boros K., Poreisz C., Chaieb L., Terney D., Paulus W. (2008). Comparatively weak after-effects of transcranial alternating current stimulation (tACS) on cortical excitability in humans. Brain Stimul..

[102] Naro A., Bramanti A., Leo A., Manuli A., Sciarrone F., Russo M., Bramanti P., Calabrò R.S. (2017). Effects of cerebellar transcranial alternating current stimulation on motor cortex excitability and motor function. Brain Struct. Funct..

[103] Miyaguchi S., Otsuru N., Kojima S., Saito K., Inukai Y., Masaki M., Onishi H. (2018). Transcranial alternating current stimulation with gamma oscillations over the primary motor cortex and cerebellar hemisphere improved visuomotor performance. Front. Behav. Neurosci..

[104] Miyaguchi S., Otsuru N., Kojima S., Yokota H., Saito K., Inukai Y., Onishi H. (2019). Gamma tACS over M1 and cerebellar hemisphere improves motor performance in a phase-specific manner. Neurosci. Lett..

[105] Miyaguchi S., Otsuru N., Kojima S., Yokota H., Saito K., Inukai Y., Onishi H. (2019). The effect of gamma tACS over the M1 region and cerebellar hemisphere does not depend on current intensity. J. Clin. Neurosci..

[106] Naro A., Leo A., Russo M., Cannavò A., Milardi D., Bramanti P., Calabrò R.S. (2016). Does Transcranial Alternating Current Stimulation Induce Cerebellum Plasticity? Feasibility, Safety and Efficacy of a Novel Electrophysiological Approach. Brain Stimul..

[107] Kuo S.H., Lin C.Y., Wang J., Sims P.A., Pan M.K., Liou J.Y., Lee D., Tate W.J., Kelly G.C., Louis E.D. (2017). Climbing fiber-Purkinje cell synaptic pathology in tremor and cerebellar degenerative diseases. Acta Neuropathol..

